# Cross-sectional evaluation of socioeconomic and clinical factors and the impact of fibromyalgia on the quality of life of patients during the COVID-19 pandemic

**DOI:** 10.1590/1516-3180.2022.0051.R2119052022

**Published:** 2022-09-12

**Authors:** Helena Trevisan Schroeder, Joana Caline Alves Cavalheiro, Edna Thaís Jeremias Martins, Patricia Martins Bock

**Affiliations:** I MSc. Biomedical, Doctoral Student, Physiology Department, Universidade Federal do Rio Grande do Sul (UFRGS), Porto Alegre (RS), Brazil.; II BSc. Nurse, Nursing Department, Faculdades Integradas de Taquara (FACCAT), Taquara (RS), Brazil.; III PhD. Nurse, Professor, Nursing Department, Faculdades Integradas de Taquara (FACCAT), Taquara (RS), Brazil.; IV PhD. Pharmacist, Professor, Nursing Department, Faculdades Integradas de Taquara (FACCAT), Taquara (RS), Brazil.

**Keywords:** Fibromyalgia, Quality of life, Complementary therapies, Cross-sectional studies, Pain, Pharmacological treatment, Fibromyalgia impact questionnaire, Self-reported quality of life

## Abstract

**BACKGROUND:**

The fibromyalgia impact questionnaire (FIQ) relates to the functional capacity, professional situation, psychological disorders, and physical symptoms, and can identify the factors that determine the impact of the syndrome and characteristics of its carriers; the higher the score, the greater the impact of fibromyalgia on the quality of life.

**OBJECTIVE:**

To evaluate the impact of fibromyalgia on the quality of life of individuals with fibromyalgia, who were categorized according to the FIQ during the coronavirus disease pandemic.

**DESIGN AND SETTING:**

A cross-sectional study was conducted at an institution of higher education in Taquara, RS, Brazil.

**METHODS:**

A quantitative study was carried out, with the application of a sociodemographic and clinical questionnaire, and the FIQ in 163 Brazilian individuals with a medical diagnosis of fibromyalgia. Data were collected using SurveyMonkey software.

**RESULTS:**

Of the female carriers, 98.2% were living in urban areas, working, and under pharmacological and complementary treatment. The FIQ results showed that seven of the 10 items had the maximum score. The items “physical function” and “feel good” had intermediate scores, and the item “missed work” had a low score. The average total score was 79.9 points, indicating that fibromyalgia had a severe impact on the participants’ lives. A severe impact of fibromyalgia was observed in 61.3% of the participants, a moderate impact in 30.7%, and a low impact in 8%.

**CONCLUSION:**

The survey findings suggest a severe impact in the majority of the Brazilian fibromyalgic population.

## INTRODUCTION

Fibromyalgia is a complex systemic disorder characterized by diffuse pain, fatigue, anxiety and depression, among other symptoms.^1^ Approximately 2.1% of the population is a carrier of fibromyalgia worldwide; however, it should be noted that regional differences can be observed.^2,3^ A prevalence of 6.1% was observed in the United States,^4^ while similar proportions to those worldwide were observed in Spain and Brazil (2.6% and 2%, respectively).^5,6^ Moreover,this syndrome is more prevalent in women.^2^

Diffuse pain is the symptom that prevails in patients with fibromyalgia; additionally, it is difficult to accurately assess its intensity, since pain is perceived subjectively and individually.^7^ The symptoms can increase according to modulating factors, such as climate change,^8^ degree of physical activity, and high stress levels,^9^ such as those experienced throughout the year 2020 with the confrontation of the global pandemic of coronavirus disease (COVID-19)^10^ by the reduction of social contact, leisure activities, financial concern, and with the health of friends and family members.^11,12^ Regarding the consequences of the syndrome, fibromyalgia has a direct influence on the mental health of the carrier, since the fewer symptoms the patient presents, the closer to a positive mental health model the patient will be.^13^

A concept linked to mental health is the quality of life, defined by the World Health Organization as an individual’s perception of their position in life, in their own context and in relation to their goals and expectations.^14^ To assess the quality of life of patients with fibromyalgia, several instruments can be used, including the fibromyalgia impact questionnaire (FIQ), which relates to the functional capacity, work situation, psychological disorders, and physical symptoms. It is a very useful tool that can identify the factors that determine the impact and collaborate to define the best treatment.^15^ However, it is often used incompletely and does not explore the categorization of the scores obtained individually by the participants. As fibromyalgia negatively impacts different aspects of the lives of individuals affected by the syndrome, it is of utmost importance to understand the profile and characteristics of its carriers and how often different impacts occur in the populations studied.

## OBJECTIVE

This study aimed to assess the impact of fibromyalgia on the lives of individuals with the syndrome during the COVID-19 pandemic as well as to map the socioeconomic and clinical factors associated with this diagnosis. It is the first study to present the Brazilian frequencies in a categorized way according to the FIQ.

## METHODS

### Study design

A cross-sectional, quantitative study was conducted, with the application of a sociodemographic and clinical questionnaire and the fibromyalgia impact questionnaire (FIQ). This study was approved as per the certificate of presentation of ethical appreciation (CAAE) (number 35691120.2.0000.8135) on August 28, 2020. The study was conducted according to the Strengthening the Reporting of Observational Studies in Epidemiology (STROBE) reporting guidelines.^16^

### Population and sample

We included Brazilian individuals (living in Brazil or not) with a medical diagnosis of fibromyalgia and older than 18 years of age. Participants who agreed to participate in the study but did not answer the questionnaire were excluded.

To calculate the sample size, the effect of fibromyalgia on the lives of patients with the syndrome was the primary outcome. As no studies were found that evaluated the ratio of severe impact on the lives of the study population, an estimated 50% of individuals suffering from a severe impact on their lives were included for the sample size calculation. A confidence level of 95% was adopted with a maximum error of 8%; additionally, the calculated sample size was 151 individuals. An additional 15% was included in the sample to minimize the possible sample losses for a total intended sample size of 173 subjects.

The participants completed digital questionnaires generated on the SurveyMonkey platform (Momentive, San Mateo, California, United States; https://pt.surveymonkey.com), from August to October 2020, during the third quarter of the COVID-19 pandemic in Brazil. The sample was selected, and access to the questionnaires was provided through social media.

The FIQ version that was validated in Brazil was applied. This questionnaire aimed to evaluate the quality of life of patients with fibromyalgia and was composed of 19 questions organized into 10 items. All the items were measured by a visual scale corresponding to values from 0 to 10 (0 = the best possible and 10 = the worst possible).^17^ To obtain the total score, the individual scores of the first three items were properly recoded by a rule of three to ten points per item; subsequently, they were added to the next seven items. If any question was left blank, the scores obtained were summed and divided by the number of questions answered.^18^ The total FIQ scores ranged from 0 to 100, where higher values indicated a greater negative impact of the syndrome, and were be classified into the following categories: low impact (< 50 points), moderate impact (50–75 points), and severe impact (> 75 points).^19^

### Statistical analysis

The Statistical Package for Social Science Professional software (version 25.0; IBM Corp., Armonk, New York, United States) was used for data analysis. The mean and standard deviation were used to describe parametric continuous variables; additionally, the median and interquartile range were used for nonparametric variables, while absolute and relative frequencies were used for categorical variables. The Shapiro–Wilk test was used to test the normality of the data; furthermore, the chi-square test was used to assess the difference in proportions between the FIQ categories.

## RESULTS

A total of 173 acceptances were obtained for participation in the survey; however, only 163 participants completed the questionnaires. The general characteristics of the participants are presented in Table 1. The questionnaires were answered by 160 women and three men, aged between 19 and 63 years.

**Table 1.** General characteristics of the study population

General characteristics	Mean/Absolute frequency	Standard deviation/Relative frequency	n
**Age (years)**	38.87	±9.05	163
**Sex**			
Male	3	(1.8)	163
Female	160	(98.2)	
**Marital status**			
Married	102	(62.6)	163
Single	43	(26.4)	
Stable union	7	(4.3)	
Divorced	11	(6.7)	
**Residence**			
Urban area	158	(96.9)	163
Rural area	5	(3.1)	
**Brazil region**			
South	19	(11.7)	163
Southeast	75	(46.0)	
Midwest	11	(6.7)	
North	8	(4.9)	
Northeast	47	(28.8)	
Outside Brazil	3	(1.8)	
**Education**			
Up to the 4^th^ grade	5	(3.1)	163
Elementary school	7	(4.3)	
High school	58	(35.6)	
Higher education	42	(25.8)	
Graduate school	51	(31.3)	
**Working**			
Yes	106	(65.0)	163
No	57	(35.0)	
**Work (hours/day)**			
4	41	(25.2)	163
6	36	(22.1)	
8	60	(36.8)	
12	26	(16.0)	

Continuous variables are expressed as the mean ± standard deviation. Categorical variables are expressed as numbers (%).

Considering the impact of fibromyalgia, 13 subjects, who were evaluated as having low impact, that is, 100% of this group, lived in urban areas. Similarly, 49 subjects with moderate impact and 96 subjects with severe impact, equal to 98% and 96% of the total in each group, respectively, also lived in urban areas.

Clinical data of the participants are presented in Table 2. The participants had a symptom onset between 7 and 50 years of age and between 13 and 52 years of age at diagnosis. The time of illness, current age, age at diagnosis, and age at symptom onset did not seem to be related to the category of impact according to the FIQ of the participants. The age of the participants with low impact, moderate impact, and severe impact was 37.08 ± 8.30 years, 39.22 ± 9.28 years, and 38.93 ± 9.09 years (P = 0.798), respectively; the age of symptom onset of participants with low impact, moderate impact, and severe impact was 29.69 ± 12.23 years, 29.14 ± 9.23 years, 28.02 ± 9.59 years (P = 0.306), respectively; the age at diagnosis of participants with low impact, moderate impact, and severe impact was 32.91 ± 8.62 years, 34.40 ± 8.48 years, and 34.14 ± 8.73 years (P = 0.895), respectively; the length of illness since diagnosis for participants with low impact, moderate impact, and severe impact was 4 (0–5) years, 3 (1–6) years, and severe 3 (1–6) years (P = 0.214), respectively; and the length of illness from the onset of symptoms for participants with low impact, moderate impact, and severe impact was 5 (3–9) years, 8 (4–16) years and 9 (4–15) years (P = 0.352), respectively.

**Table 2.** Clinical data of the study population

Clinical characteristics	Mean/Absolute frequency	Standard deviation/Relative frequency	n
**Age at symptom onset (years)**	28.5	±9.7	163
**Age at diagnosis (years)**	34.1	±8.6	160
**Time of diagnosis (years)**	3	(0-6)	160
**Time of illness (years)**	8	(4-15)	163
**Physicians who made the diagnosis**			
Rheumatologists	103	(63.2)	163
General Practitioner	12	(7.4)	
Neurologist	14	(8.6)	
Orthopedist	27	(16.6)	
Others	7	(4.3)	
**Physicians who performed the treatment**			
Rheumatologists	55	(33.7)	163
General Practitioner	8	(4.9)	
Neurologist	4	(2.5)	
Orthopedist	12	(7.4)	
Psychologist or Psychiatrist	11	(6.7)	
Others	6	(3.7)	
More than one	57	(35.0)	
None	10	(6.1)	
**Event that triggered the FM symptoms**			
Depression	27	(16.6)	163
Occupational disease	7	(4.3)	
Emotional trauma	35	(21.5)	
Genetic inheritance	7	(4.3)	
Physical trauma (accident/fall)	9	(5.5)	
Change in lifestyle	6	(3.7)	
Medication use	2	(1.2)	
Surgery	5	(3.1)	
did not know	65	(39.9)	
**FM symptoms**			
Tiredness/Fatigue	156	(95.7)	163
Localized pain	151	(92.6)	163
Sleep disturbances	144	(88.3)	163
Memory loss	136	(83.4)	163
Joint stiffness	124	(76.1)	163
Anxiety	143	(87.7)	163
Difficulty concentrating	138	(84.7)	163
Tingling	123	(75.5)	163
Others	47	(28.8)	163
**Increasing symptoms aspects**			
Exaggerated physical exertion	114	(69.9)	163
Stress	146	(89.6)	163
Nighttime	45	(27.6)	163
Emotional state	144	(88.3)	163
Others	28	(17.2)	163
**Period of de day with major pain**			
Morning	72	(44.2)	163
Evening	17	(10.4)	
Night	74	(45.4)	
**FM impact self-reported**			
Low	1	(0.6)	163
Moderate	52	(31.9)	
Severe	110	(67.5)	
**Associated disorders**			
RSI	47	(28.8)	163
Musculoskeletal disorder	17	(10.4)	163
Lupus	7	(4.3)	163
Chronic fatigue syndrome	47	(28.8)	163
None	76	(46.6)	163

FM = fibromyalgia; RSI = repetitive strain injury. The time of illness refers to the age at symptom onset. Continuous variables are expressed as mean ± standard deviation or median [interquartile range (p25–75)]. Categorical variables are expressed as numbers.

Regarding the symptoms of fibromyalgia, it is important to note that the impact of fibromyalgia does not seem to be related to symptoms, namely localized pain (low - 13, 100%; moderate - 45, 90%; and severe - 93, 93%; P = 0.458), memory loss (low - 11, 84.6%; moderate 38, 76.0%; and severe - 87, 87.0%; P = 0.231), tingling (low - 7, 53.8%; moderate - 37, 74.0%; and severe - 79, 79.0%; P = 0.134) and tiredness or fatigue (low - 11, 84.6%; moderate - 47, 94.0%; and severe - 98, 98.0%; P = 0.063), while there was a higher frequency of individuals with sleep disturbances (low - 9, 69.2%; moderate - 41, 82.0%; and severe - 94, 94.0%; P = 0.008), difficulty concentrating (low - 10, 76.9%; moderate - 35, 75.0%; and severe 93, 93%; P = 0.001), joint stiffness (low - 11, 84.6%; moderate - 29, 58.0%; and severe - 84, 84.0%; P = 0.002), and anxiety (low - 9, 69.2%; moderate - 40, 80.0%; and severe - 94, 94.0%; P = 0.005) in those most impacted by fibromyalgia.

Notably, when the results of the impact self-reported by the participants and the one obtained by the FIQ questionnaire were cross-checked, there was better agreement on the greatest impact, where 83 subjects in the severe group (83%) declared themselves to be in the same group (the other 17 considered themselves to have moderate impact), 26 subjects classified by the FIQ as moderate considered moderate impact (52%), the remaining 23 subjects (46%) considered their impact as severe, and 1 (2%) subject considered their impact as low, while the participants evaluated as having low impact indicated moderate (9, 69.2%) or severe impact (4, 30.8%).

Regarding other associated disorders, repetitive strain injury (28.8%), chronic fatigue syndrome (28.8%), musculoskeletal disorder (10.4%), and lupus (4.3%) were observed, with 46.6% of the participants having only fibromyalgia; additionally, among these participants, 30.8% had a low impact by fibromyalgia, 54.0% had a moderate impact, and 45.0% had a severe impact, according to the FIQ.

Data related to the treatments used by the participants are presented in Table 3. Regarding non-pharmacological treatments, most participants used some non-pharmacological support treatment, while 47.9% did not use any treatment (among them, 67.9% had a severe impact).

**Table 3.** Fibromyalgia treatment of the study participants

Treatment characteristics	Absolute frequency	Relative frequency	n
**Non-pharmacological treatment**				
Physiotherapy/Massage	21	(12.9)	163163
Psychologist/Psychiatrist	17	(10.4)	
Acupuncture/Auriculo therapy	5	(3.1)	
Other alternative treatments	42	(25.8)	
None	78	(47.9)	
**Pharmacological treatment**				
Antidepressants	64	(39.3)	163
Antidepressants and analgesics	24	(14.7)	
Antidepressants and muscle relaxants	27	(16.6)	
Antidepressants and anti-inflammatory drugs	3	(1.8)	
Muscle relaxants	8	(4.9)	
Other drug combinations	7	(4.2)	
None	30	(18.4)	
**Physical exercise**			
Walking/Running/Cycling	30	(18.4)	163
Pilates/Yoga	16	(9.8)	
Weightlifting	18	(11.0)	
Other modalities	21	(12.9)	
More than one	16	(9.8)	
None	62	(38.0)	
**Exercise frequency**				
Up to 2 times a week	40	(24.5)	163
Up to 4 times a week	41	(25.2)	
Up to 6 times a week	24	(14.7)	
None	58	(35.6)	

Categorical variables are expressed as numbers.

The pharmacological treatments used by the participants included only antidepressants (39.3%, of whom 6.3% belonged to the low-impact group of fibromyalgia, 32.8% belonged to moderate-impact group, and 60.9% belonged to the severe-impact group), antidepressants and muscle relaxants (16.6%, low 4.2%, moderate 25.0%,and 70.8% severe), antidepressants and analgesics (14.7%, 0.0% low, 37.0% moderate, and 63.0% severe), muscle relaxants only (4.9%, 25% low, 25% moderate, and 50% severe), other drug combinations (4%, 3% low, 14.3% moderate, and 85.7% severe), antidepressants and anti-inflammatory drugs (1.8%, 33.3% in each category), and no medication (18.4%, 16.7% low, 30.0% moderate, and 53.3% severe).

Regarding the performance of physical exercise, analyzing the categories of impact of fibromyalgia on the lives of the participants, 45.0% of the members of the severe impact group did not perform any kind of physical exercise, while the others were divided into walking, running, or cycling (21.0%), other modalities (14.0%), pilates or yoga (8.0%), weight training (8.0%), and more than one modality (4.0%).

Table 4presents the results of the FIQ. The results show that of the 10 items, 7 items (do work, pain, fatigue, rested, stiffness, anxiety, and depression) had the maximum score of 10 points, demonstrative of a worse condition relative to each item. The items of physical function and feeling good were scored with intermediate scores (5.33 and 7.14, respectively), while in the item missed work, we could consider the low score obtained (2.86). The median total score was 79.9 points, with an interquartile range of 66.7–85.9, indicating that fibromyalgia has a severe impact on the lives of the participants. A severe impact of fibromyalgia was observed in 61.3% of the participants, moderate impact in 30.7%, and low impact in 8% of the participants.

**Table 4.** Scores in the fibromyalgia impact questionnaire (FIQ)

FIQ items	Median/Absolute frequency	Interquartile range/Relative frequency	n
Physical function	5.33	(3.83–6.67)	
Feel good	7.14	(5.71–8.57)	
Missed work	2.86	(0–7.14)	
Do job	10	(7–10)	
Pain	10	(8–10)	
Fatigue	10	(10–10)	
Rested	10	(8–10)	
Stiffness	10	(8–10)	
Anxiety	10	(7.5–10)	
Depression	10	(6.5–10)	
**FIQ scores**	**79.9**	**(66.7–85.9)**	**163**
**FIQ categories**			
Low	13	(8.0)	163
Moderate	50	(30.7)	
Severe	100	(61.3)	

Continuous variables are expressed as mean ± standard deviation or median [interquartile range (p25–75)]. Categorical variables are expressed as numbers.

## DISCUSSION

The present study evaluated the impact of fibromyalgia on the lives of its carriers and investigated the socioeconomic and clinical factors present. This is the first study with a sample of the Brazilian population to measure the ratio among the categories of the FIQ. The results indicate a severe impact on the lives of the individuals with fibromyalgia, not only by the high result obtained in the FIQ score, but also by a large number of individuals in the severe impact category.

This research pointed to a total score of 79.9 for the FIQ, a value similar to that found in the literature by Martinez et al., who obtained a score of 70.3.^20^ Even higher scores can be found; of the 10 items evaluated, nine had high scores.^21^ This fact suggests that most carriers suffer from a severe impact of the syndrome. It is important to emphasize that the more pain the patient reports, the higher the FIQ score and consequently, the worse the quality of life of that individual will be.^22^ Contrastingly, patients who have low impact due to fibromyalgia have better acceptance of their pain than those with severe impact.^23^ An important observation to be made is that when the participant was asked about the impact of fibromyalgia on their life, 67.5% indicated having a severe impact, which was not far from the results found by the FIQ, which showed that 61.3% of participants have severe impact. This demonstrated an accurate self-perception of the participant with respect to their condition. In addition, notably, there seems to be a link between self-awareness related to the syndrome and management of the crises generated by it with the FIQ scores, which are lower in carriers who have this control.^6^

High frequencies of depressive and anxiety symptoms are also found in carriers of fibromyalgia,^24^ noting that these symptoms occur in greater intensity in those in whom fibromyalgia causes a severe impact.^25^ In the present study, no analysis was performed with specific questionnaires for depression and anxiety; however, in the clinical questionnaire, more than 70% of the participants reported having memory loss, even though no relationship was observed with the fibromyalgia impact group. In addition, a higher frequency of anxiety, difficulty in concentrating, and sleep disturbances was observed among those with the highest impact. Regarding the emotional aspects of the syndrome, we observed a high proportion of individuals participating in this study who reported not knowing the origin of the onset of their symptoms. However, of those who did know, 65 participants (39.4% of the total) reported an emotional relationship, either depression or emotional trauma, and most of them were individuals categorized by the FIQ as being severely impacted by fibromyalgia.

The data collection period corresponded to the third quarter of the COVID-19 pandemic in Brazil. This could be related to the high severity of fibromyalgia found in the study subjects. Therefore, besides the fact that the presence of the viral infection itself (a parameter not evaluated in the clinical questionnaire applied) seems to worsen all domains of the FIQ in fibromyalgia patients,^26^ the potential aggravation of stress and fear caused by the pandemic on the symptoms faced by fibromyalgia sufferers is discussed.^27^ Hausmann et al. observed substantial changes in the employment status in their study sample and linked this to decreased access to fibromyalgia health care and treatment during the pandemic.^28^

With respect to work, a study conducted in 2020 in Saudi Arabia found a high prevalence of fibromyalgia sufferers among healthcare workers.^29^ The frontline healthcare workers for COVID-19 had to deal directly with an overload of work, being drastically affected by emotional stress, causing depression and anxiety.^30^ These factors are related to the management of fibromyalgia and, as previously mentioned, with a high frequency in the group severely impacted by the syndrome. Although the present study did not access the participants’ areas of expertise, this could be a factor that may have influenced the results obtained. In addition to those who worked directly with healthcare in the pandemic, the confinement situation adopted by several countries forced many patients to discontinue their treatments^31^ and exacerbated the main symptoms of fibromyalgia.^32^ Moreover, some authors found no influence of the pandemic on the clinical manifestations of fibromyalgia,^33^ keeping this question open.

The fact that more than 90% of the participants reside in an urban area is in agreement with a previous study that showed that a greater number of individuals with fibromyalgia live in urban areas, with a prevalence ranging between 0.69% and 11.4%, higher than in a rural area that showed a prevalence between 0.6% and 5.2% of the population.^3^ Corroborating the findings of the present study, Martinez et al., in a Brazilian study, selected patients with fibromyalgia according to the degree of severity obtained by the FIQ, and showed that there seems to be no relationship between the degree of severity and the patient’s age, age at onset of symptoms, family income, education, or other diseases associated with fibromyalgia.^34^

In this research, the number of female participants was the majority, which corroborates with other studies that also demonstrate a higher number of women with the syndrome for example, the study conducted by Tangenet al., in which 97% of the sample were women.^23^ Additionally, Cabo-Meseguer et al. also observed a higher number of women (4.3%) than men (0.49%) with fibromyalgia.^2^ Our findings showed that most of the participants resorted to non-pharmacological interventions, mainly physiotherapy or therapeutic manipulation. A systematic review involving different musculoskeletal diseases of chronic pain, including fibromyalgia, demonstrated a positive effect of myofascial release when compared to placebo treatment on pain frequency and intensity, as well as the level of functionality and quality of life.^35^ However, a more recent systematic review focused on patients with fibromyalgia showed that the technique showed no improvement in the outcomes of pain, FIQ, and quality of life.^36^ Additionally, although a high adherence to acupuncture has not been found in the present results, this therapy proves to be very efficient for pain reduction^37^ and pain threshold increase^38^ among the non-pharmacological treatment modalities.

Another category of non-pharmacological supportive treatment used by some of the participants was physical exercise, which demonstrates an improvement of fibromyalgia symptoms and mainly imparts a willingness to perform daily activities.^39^ It has been shown that training with stretching exercises, strength training, and aerobic training for at least 60 min, 3 times a week, can improve the patient’s condition^40^ and that walking brings benefits in the quality of sleep.^41^ Even an umbrella systematic review confirmed an improvement in pain, quality of life, physiological function, and psychological function of fibromyalgia patients by the practice of physical exercise.^42^

Among the medications used today are those that can modulate some specific neurotransmitters, such as noradrenaline, serotonin, gamma-aminobutyric acid, opioid receptors, and calcium channel blockers, among others.^43^ Moreover, although we did not assess which medications are part of the treatment of the interviewed individuals, we obtained results that show that most of the interviewed individuals use at least one drug combination.

A limitation of this study is the lack of use of a comparative tool for general quality of life measurements. In addition, although the study is quite comprehensive from a regional point of view, it may have a search bias, since, possibly, patients impacted by their condition will be concerned about participating in research. Likewise, the fact that the subjects filled out the questionnaires themselves may have generated differences in the interpretation of the questions and collection of the answers. Moreover, as previously discussed, the period chosen for data collection may have increased the scores obtained because of the COVID-19 pandemic, and future studies are essential to visualize the consequent effects.

## CONCLUSION

In the evaluated sample, we observed a higher frequency of the severe impact category, as well as a higher FIQ score during the observation during the COVID-19 pandemic, which demonstrates a poor quality of life in these individuals. In addition, the majority of fibromyalgia patients are women who live in urban areas, work, and use pharmacological and complementary treatments. A higher frequency of anxiety, difficulty concentrating, and sleep disturbances were related to a severe impact. Moreover, even if individuals practice some physical activity, fibromyalgia is observed to severely affect their lives.
